# Influence of Polymer Processing on the Double Electrical Percolation Threshold in PLA/PCL/GNP Nanocomposites

**DOI:** 10.3390/s22239231

**Published:** 2022-11-27

**Authors:** Nour-Alhoda Masarra, Jean-Christophe Quantin, Marcos Batistella, Roland El Hage, Monica Francesca Pucci, José-Marie Lopez-Cuesta

**Affiliations:** 1 Polymers Composites and Hybrids (PCH), IMT Mines Ales, 30100 Ales, France; 2 LMGC, IMT Mines Ales, Montpellier University, CNRS, 30319 Ales, France; 3 Laboratory of Physical Chemistry of Materials (LCPM), PR2N (EDST), Faculty of Sciences II, Lebanese University, Campus Fanar P.O. Box 90656, Lebanon

**Keywords:** double electrical percolation, electrical conductivity, co-continuous structure, 3D printing, bio nanocomposite blends, PLA, PCL, GNP

## Abstract

For the first time, the double electrical percolation threshold was obtained in polylactide (PLA)/polycaprolactone (PCL)/graphene nanoplatelet (GNP) composite systems, prepared by compression moulding and fused filament fabrication (FFF). Using scanning electron microscopy (SEM) and atomic force microscopy (AFM), the localisation of the GNP, as well as the morphology of PLA and PCL phases, were evaluated and correlated with the electrical conductivity results estimated by the four-point probe method electrical measurements. The solvent extraction method was used to confirm and quantify the co-continuity in these samples. At 10 wt.% of the GNP, compression-moulded samples possessed a wide co-continuity range, varying from PLA55/PCL45 to PLA70/PCL30. The best electrical conductivity results were found for compression-moulded and 3D-printed PLA65/PCL35/GNP that have the fully co-continuous structure, based on the experimental and theoretical findings. This composite owns the highest storage modulus and complex viscosity at low angular frequency range, according to the melt shear rheology. Moreover, it exhibited the highest char formation and polymers degrees of crystallinity after the thermal investigation by thermogravimetric analysis (TGA) and differential scanning calorimetry (DSC), respectively. The effect of the GNP content, compression moulding time, and multiple twin-screw extrusion blending steps on the co-continuity were also evaluated. The results showed that increasing the GNP content decreased the continuity of the polymer phases. Therefore, this work concluded that polymer processing methods impact the electrical percolation threshold and that the 3D printing of polymer composites entails higher electrical resistance as compared to compression moulding.

## 1. Introduction

Nowadays, conductive polymer composites (CPCs) based on biodegradable matrices are required in various applications, such as electromagnetic interference shielding (EMI), sensors, wearable and portable devices, conductors, and capacitors [1,2,3,4,5] because they are light, cost-effective, eco-friendly, and easily processed. However, it is important to manufacture these composites with a low content of conductive fillers to avoid problems, such as low mechanical properties, high costs, and high melt viscosities [6]. One of the most effective methods to reduce the electrical percolation threshold is to add the conductive fillers to an immiscible polymer blend that possesses a co-continuous structure. In this method, conductive fillers are selectively located in one polymer phase or at the interface [7] and can therefore yield what is known as “double percolation”. The latter refers to the percolation of the filler within one phase of the polymer blend (first percolation) that itself percolates in the blend (double percolation) [8]. The selective localisation of nanofillers is affected by different factors, such as the kinetic parameters that affect their migration and their final localisation. Examples of these parameters are: compounding sequence [9,10,11], melt viscosity of polymer components [10,12,13], shear rate [14,15], and melt compounding time [7,16,17,18]. Thermodynamic factors, such as the surface energy of the nanofiller and the two polymer components, greatly affect this preferential positioning of the nanofillers in the polymer blends [19].

Due to their tendency to be selectively distributed in polymer blends, carbon fillers have been extensively used to create the double electrical percolation in the polymer composites. In this context, the most implemented carbon fillers are carbon nanotubes [7,20,21,22,23] and carbon black [24,25,26,27,28]. Graphene was also used for this purpose in two previously published contributions. Kou et al. [29] succeeded to interfacially localise GNPs in a co-continuous polymer blend constituted of PLA and poly (ethylene-co-vinyl acetate) (EVA). They prepared a ternary composite through a two-step compounding sequence where the PLA/GNP masterbatch was first prepared via solution blending, and then melt compounded with EVA. In the second step, the GNPs are transferred to the thermodynamically favoured phase (EVA) to be trapped at the interface. This resulted in an ultralow percolation threshold of 0.048 wt.% and an electrical conductivity of 10^−3^ S·m^−1^ at 0.5 wt.% of the GNPs. Using the same experimental protocol, Bai et al. [30] introduced GNPs into a blend composed of co-continuous PLA and polystyrene (PS). These authors were also able to trap graphene at the interface of the blend, although it has preference to PS. The double electrical percolation obtained led to an electrical conductivity of 10^−6^ S·cm^−1^ at 0.5 wt.% of the GNPs.

There are different methods to manufacture test samples of conductive carbon-based polymer nanocomposites. Conventional processing techniques, such as compression moulding and more recent ones such as fused filament fabrication (FFF), can be used for this purpose. There is only one published work regarding the impact of the two mentioned processes on the double electrical percolation. In this regard, Bertolini et al. [31] manufactured co-continuous poly(vinylidene fluoride) (PVDF)/thermoplastic polyurethane (TPU) (50 vol.%/50 vol.%) filled with CB-polypyrrole. The filler was preferably localized in the PVDF phase. Their results demonstrated that at the same filler content, the samples possessing a co-continuous structure have higher conductivities as compared to the ones with dispersed morphologies. Thus, the 3D printing of the co-continuous composites gave a material that can be used for electrically conductive applications. For example, 3D-printed co-continuous composite containing 6.77 vol.% of filler presented an electrical conductivity of 4.14 S·m^−1^.

This work is a pioneer in studying the influence of compression moulding and FFF on the double electrical percolation threshold in graphene-based polymer blend composites. PLA/PCL/GNP nanocomposites were prepared using twin-screw extrusion for this objective. The range of blend ratios in which the existence of the co-continuous morphology, accompanied by the double electrical percolation threshold, was determined for compression-moulded samples. Rheological and thermal tests were performed to evaluate if the co-continuous microstructure can entail superior viscoelastic and thermal stability characteristics. After combining the information from the microstructural and the electrical properties, the most interesting co-continuous composition was revealed. Moreover, the impact of the variation of graphene percentage and some processing parameters on the microstructure were investigated. Finally, the possibility of manufacturing electrically conductive graphene co-continuous nanocomposites using the FFF technique was verified for the first time. Hence, this work aims to represent the first attempt of creating electrically conductive graphene biodegradable nanocomposites having double electrical percolation.

## 2. Materials and Methods

### 2.1. Materials

PLA Ingeo^TM^ Biopolymer 2003D was provided from NatureWorks (Minnetonka, MN, USA). It has a melt flow rate of 6 g/10 min (210 °C/2.16 kg), a glass transition temperature of 63 °C, melting temperature of 156 °C, and a density of 1.24 g·cm^−3^. PCL grade Capa^TM^ 6800 was purchased from Perstorp UK Ltd. (Warrington, UK). This grade has a melt flow rate in the range of 2.01–4.03 g/10 min (160 °C/5 kg), a melting temperature in the 58–60 °C range, and a glass transition temperature of −60 °C.

M5 grade graphene nanoplatelets purchased from XG Sciences Inc., Lansing, MI, USA is characterized by: an average lateral dimension of 5 μm, a thickness in the range of 6–8 nm, a specific surface area of 120–150 m^2^·g^−1^, a high electrical conductivity of 10^7^ S·m^−1^, and a density of 2.2 g·cm^−3^.

### 2.2. Samples’ Preparation

#### 2.2.1. Nanocomposite Blends Preparation

**Figure 1:**
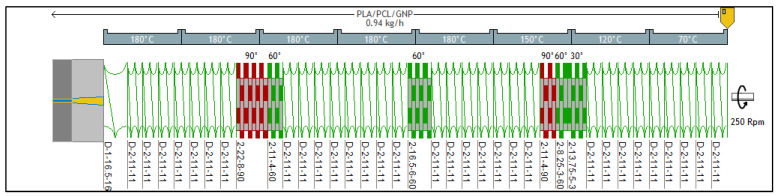
Screw profile and conditions set during PLA/PCL/GNP nanocomposites processing (picture from Ludovic^®^ software).

Twin-screw extrusion (Process 11, Thermo Scientific, MA, USA) was used to mix PLA, PCL, and GNP together. The parameters set in this process are demonstrated in Figure 1. The materials were dried overnight prior to the extrusion experiments (PLA and PCL were vacuum dried at 60 °C and 40 °C, respectively, whereas GNP powder was dried in a conventional oven at 80 °C). Table 1 summarises the three main compositions of the extruded nanocomposites and the reason behind preparing each.

For the purpose of using several storage moduli and complex viscosity-based models that help in the theoretical prediction of the co-continuity, a twin-screw extrusion of PCL/GNP composites containing graphene percentage varying between 10 wt.% and 20 wt.% was carried out. The screw profile was not changed as compared to the previous batch (Figure 1). The parameters set in these experiments are: a flow rate of 0.94 kg.h^−1^, a screw speed of 200 rpm, and a temperature profile of 60 °C (feeding zone)–70 °C–75 °C–80 °C–80 °C–85 °C (all the remaining barrel zones until the die zone). 

#### 2.2.2. Compression Moulding

The vacuum dried nanocomposites and pure PLA pellets were compression moulded using a laboratory hot press (LAB 800 PA, PINETTE PEI, France) by maintaining them for 10 min at 180 °C, a rate of 30 °C·min^−1^, and a load of 500 kN. Sample demoulding was achieved after cooling down to 80 °C at a rate of 30 °C·min^−1^ using a water-cooling system. The prepared samples were disks of 25 × 6 mm intended for melt-shear rheological tests, scanning electron microscopy (SEM), and atomic force microscopy (AFM). Moreover, square plates of 100 × 100 × 3 mm, from which bars of 30 × 5 × 3 mm were cut for the electrical resistance tests, were also manufactured. For the viscosity-based models, PCL/GNP disks were prepared by applying a load of 200 kN at 120 °C for 10 min. Afterwards, the cooling step took place by decreasing the temperature to 50 °C at a rate of 20 °C·min^−1^ before samples demoulding. The overall time of compression moulding of all the samples was 35 min. Yet, several PLA65/PCL35/10 wt.% GNP samples were prepared during 10, 20, and 35 min to study the impact of the annealing time increase on the co-continuity. 

#### 2.2.3. Filament Preparation

The nanocomposite pellets were used to feed a mini single-screw extruder (3devo, Utrecht, Netherlands) to prepare calibrated filaments with a diameter of 2.83 mm. The obtained filament was used as an input material in the 3D printing process. The filament extrusion parameters are: a speed of 5 rpm and a temperature profile of 165 °C (feeding zone)–190 °C–200 °C–195 °C. 

#### 2.2.4. Fused Filament Fabrication

An A4v3 FFF printer (3ntr, 28047 Oleggio, NO, Italy) was used to print the manufactured filaments into bars and disks for the electrical resistance measurements and the microscopy investigations, respectively. For the aim of comparison, the manufactured samples have the same dimensions as the compression-moulded ones and the applied processing parameters are: a nozzle diameter of 0.8 mm, a printing speed of 60 mm.s^−1^, a primary layer height of 0.2 mm, a raster angle of +45°/−45°, a nozzle temperature of 180 °C, and a heated platform temperature of 60 °C. The 3D-printed samples are: PLAx/PCLy/10 wt.% GNP (x varies between 80 and 30 wt.% with respect to the total weight fraction of 90 wt.% of polymers).

### 2.3. Characterisation Techniques

#### 2.3.1. Scanning Electron Microscopy (SEM)

The morphologies of the compression moulded and 3D-printed samples were analysed along their transversal section using a scanning electron microscope (SEM) (FEI Quanta 200, FEI Company, Hillsboro, OR, USA) at an acceleration voltage of 10 kV. A cryo-ultramicrotome (Leica EM UC7, Nanterre, France) was used to smooth the surface to obtain a better contrast and quality of observations with SEM. The obtained images were produced via back scattered mode (BSE) and secondary electron (SE) beam modes. A particular preparation was performed before the SEM observation of the 3D-printed PLA65/PCL35/10% GNP composite. The sample was immersed in toluene at room temperature for 3 h to dissolve the PCL phase, followed by washing to remove the dissolved fraction. The solvent etching technique was used especially for this sample due to the difficulty in differentiating between both phases when the observations took place directly. The reason is the high roughness of the surface and the high content of PLA. This problem did not exist for 3D-printed samples containing dispersed PLA phase in the continuous PCL matrix. 

#### 2.3.2. Atomic Force Microscopy (AFM)

An atomic force microscope (AFM, MFP-3D infinity, Asylum Research, Oxford instruments, Abingdon, UK) was used to confirm the complete localisation of GNP in the PCL phase. The investigations were carried out on the cryo-fractured smooth transversal sections of each sample. The images were obtained in a bimodal tapping mode (also known as Amplitude Modulation–Frequency Modulation mode (AM–FM) mode [32]) under ambient conditions using AC160TSR3 tip at a scan rate of 1 Hz. The topography (height), phase (phase shift between excitation and response of the cantilever), and elastic modulus images were recorded and analysed to have full understanding of the results.

#### 2.3.3. Solvent Extraction Method

Several methods have been extensively used to determine the co-continuity range, including rheology [33,34,35,36], microscopy [33,37,38,39,40], and solvent extraction [41,42,43,44,45,46,47,48,49,50,51,52,53]. Solvent extraction experiments are the simplest and most popular technique to detect the co-continuity, and they require at least one of the polymer phases to be selectively removed from the sample. In this work, PCL was chosen to be extracted and not the PLA phase, since the latter is more solvent resistant and cannot be removed without damaging the PCL phase. Therefore, the PCL-containing graphene phase was extracted (the complete selective localisation of graphene in the PCL phase is proved in the first section of the results and discussion). Compression-moulded bar-shaped samples (length: 3 cm, width: 6 mm, thickness: 3 mm) with various compositions were selected for these experiments. Each sample was immersed in 5 mL of toluene in a closed test tube for 5 h and 30 min at 60 °C (the toluene was renewed every 2 h) to selectively extract the completely dissolved PCL phase. The complete extraction of the graphene filled PCL phase was verified by the darkness of the extracted solution. Before weighing, the samples were dried in an oven at 40 °C to completely evaporate the solvent. After the complete dissolution of the graphene-containing PCL phase, there exist two cases [54]:

1. The extraction left the sample compact (no fragmentation into small pieces);
  PLA is 100% continuous and the PCL continuity is quantified by the weight fraction of PCL that is extracted according to Equation (1):
  (1) $$\mathrm{PCL}\;\mathrm{continuous}\;\mathrm{fraction}\;\left(\%\right)=\frac{m_{2}}{m_{1}}\times 100,$$
  where, *m*_1_ is the mass of the graphene-filled PCL phase before the solvent dissolution (mg) ($m_{1}=m_{\mathrm{total}\;\mathrm{before}\;\mathrm{the}\;\mathrm{solvent}\;\mathrm{extraction}}\times \left(\mathrm{wt}.{\%}_{\left(\mathrm{PCL}\;+\;\mathrm{GNP}\right)}\right)$;
  *m*_2_ is the mass of graphene-filled PCL phase after the solvent dissolution (mg) ($m_{2}=m_{\mathrm{total}\;\left(\mathrm{before}\;\mathrm{the}\;\mathrm{solvent}\;\mathrm{extraction}\right)}-{\;m}_{\mathrm{total}\;\left(\mathrm{after}\;\mathrm{the}\;\mathrm{solvent}\;\mathrm{extraction}\right)})$;
  This is the case of samples containing high PLA percentage;
2. The sample fell apart:
  PCL is 100% continuous and the PLA continuity is represented by the biggest compact PLA piece released at the end of the extraction. The percentage of continuity of PLA in this case is calculated according to Equation (2):
  (2) $$\mathrm{PLA}\;\mathrm{continuous}\;\mathrm{fraction}\;\left(\%\right)\;=\;\frac{m^{\prime }}{m^{\prime \prime }}\times 100,$$
  where, *m*^’^ is the mass of the biggest part of PLA (mg);
  *m*^″^ is the mass of PLA in the sample before the solvent dissolution (mg) ($m^{\prime \prime }=m_{\mathrm{total}\;\mathrm{before}\;\mathrm{the}\;\mathrm{solvent}\;\mathrm{dissolution}}\times \left(\mathrm{wt}.{\%}_{\left(\mathrm{PLA}\right)}\right)$
  In the case of the co-continuous samples and samples containing dispersed PLA nodules in the PCL phase, the samples exhibit a fully dispersed PLA structure and a complete disintegration after the dissolution of the PCL phase where the mass of the biggest PLA part is too low to yield a negligible percentage of continuity of PLA.

#### 2.3.4. Electrical Resistance Measurements

As described by our previous work [55], a four-point probe measurement technique was used to measure the resistance of both the compression-moulded and 3D-printed bars. Measurements took place on 10 bar-shaped samples that were conditioned for at least 48 h under controlled temperature (25 °C) and humidity (50%) conditions. Each test specimen was tested at a voltage of 5 V by using a direct current (DC) power supply (Topward, TPS-4000). The voltage was measured between the two internal copper electrodes using a digital multimeter (HM 8011, HAMEG Instruments, Mainhausen, Germany) and the current flow was measured between the two external copper electrodes using a multimeter (MX 579 Metrix, ITT instruments, New York, NY, USA). These four copper electrodes were identical and equidistant. Only one surface of each sample was in contact with the electrodes. After knowing the voltage and the current, the electrical volume resistivity ρ of each sample was calculated according to Equation (3):

(3) $$\rho =\frac{R\;\times \;S}{L},$$

where R is the electrical resistance (Ω) (calculated using Ohm’s law), S is the cross-sectional area (m^2^) (equal to the width of the sample multiplied by its thickness), and L corresponds to the distance between the internal electrodes and is equal to 3.7 mm.

#### 2.3.5. Rheological Measurements

The complex viscosity and the storage modulus of the PLA, PLA/GNP, PCL/GNP, and PLA/PCL/GNP composites were evaluated using a rotational rheometer (MCR 702, Anton Paar, Graz, Austria) by performing oscillatory shear mode measurements using parallel plates geometry (25 mm diameter). At least one night prior to testing, the samples were dried in a vacuum oven at 50 °C. After verifying the thermal stability of all the samples at 180 °C and under constant flow of nitrogen gas to prevent the degradation of the samples, the strain sweep tests were performed at 628 rad·s^−1^ in order to determine the upper limit of the linear viscoelastic domain. Finally, frequency sweep tests between 0.01 and 628 rad·s^−1^ were carried out at a shear strain percentage of 1% for PLA, 0.001–0.06% for PCL/GNP, and 0.006% for PLA/PCL/GNP. 

#### 2.3.6. Differential Scanning Calorimetry (DSC)

A PerkinElmer Diamond differential scanning calorimeter (DSC) was used mainly to estimate the percentage of crystallinity of both the PLA and PCL in the pure polymers and the composites. First, the samples were weighed and sealed in aluminium pans (10 mg). Then, they were heated from −70 °C to 200 °C, held for 5 min to eliminate the previous thermal histories, cooled down to −70 °C, and finally reheated to 200 °C. The heating rate was chosen to be 10 °C·min^−1^ and the cooling rate was 5 °C·min^−1^. The crystallisation temperature, T_c_, of PLA and the melting temperatures, T_m_, of both polymers were determined from the peak values of the respective exotherms and endotherms. The degree of crystallinity of PLA and PCL in the samples (X_c_) was determined by using Equation (4):

(4) $$X_{c}=100\times \frac{\Delta H_{m}-\Delta H_{c}}{\Delta H_{m}^{{}^{\circ}}\cdot W},$$

where $\Delta H_{m}$ is the enthalpy of fusion of each polymer, $\Delta H_{c}$ is the enthalpy of crystallisation of PLA, $\Delta H_{m}^{{}^{\circ}}$ is the enthalpy of 100% crystalline polymer ($\Delta H_{m\left(\mathrm{PLA}\right)}^{{}^{\circ}}$= 93.7 J·g^−1^ and $\Delta H_{m\left(\mathrm{PCL}\right)}^{{}^{\circ}}$= 135.44 J·g^−1^) [56,57], and *W* is the weight fraction of each polymer in the blend and the composites.

#### 2.3.7. Thermogravimetric Analysis (TGA)

To correlate the sample microstructure to the thermal stability of the composites, thermogravimetric analysis (TGA) tests were accomplished. For these tests, 10 mg of each sample were subjected to heating from room temperature to 900 °C at a rate of 10 °C·min^−1^ in the presence of nitrogen gas (40 mL·min^−1^). This step followed an isothermal step for 30 min at room temperature. After recording the weight loss as a function of temperature, the following parameters were determined: the experimental percentage of char yield at 600 °C, the thermal onset degradation temperature at 15 wt.% of mass loss (T_onset_), the maximum degradation temperatures (T_max_) of PLA and/or PCL peaks, and the total mass loss over the entire temperature range.

## 3. Results and Discussion

### 3.1. Selective Localisation of Graphene in the PCL Phase in Compression-Moulded Samples

Studying the evolution of the microstructure in the compression-moulded samples is of great interest. This is related to its strong influence on the electrical conductivity of the composites that can be boosted when the co-continuous structure exists thanks to the double electrical percolation phenomenon. Since the selective localisation of the nanofillers in one polymer phase is an indispensable condition for the occurrence of the double electrical percolation, it is necessary to verify it in the PLA/PCL/GNP compositions. This selective localisation in polymer blends prepared by melt mixing is affected by various physical and processing parameters, such as the absolute viscosity, the viscosity ratio of the polymers, the elasticity of the polymers, the interfacial tension among the constituents, and the mixing parameters [58]. However, the complex viscosity ratio can be considered as one of the main factors that affects the phase morphology and the location of the fillers in the polymer blend composites [59]. 

**Figure 2:**
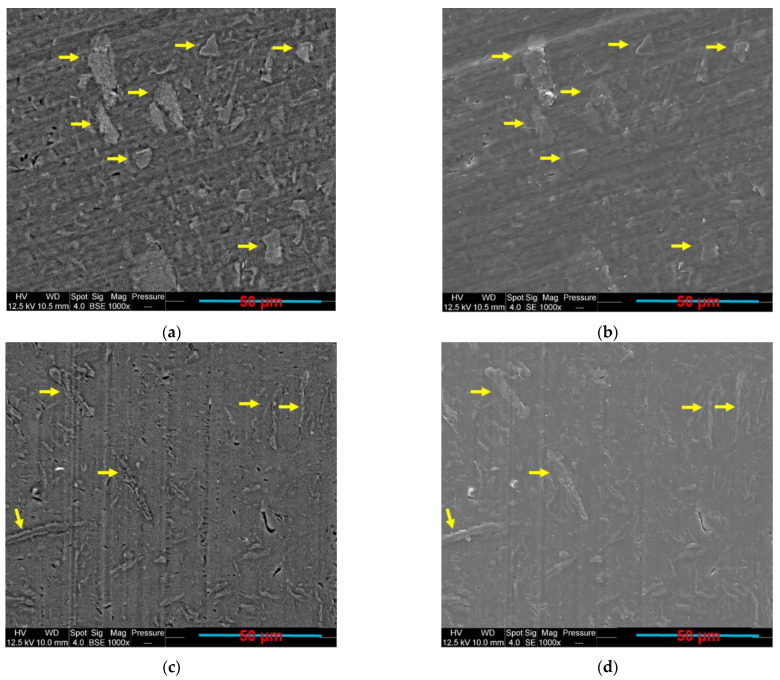
SEM BSE and SE images of (**a**,**b**) PCL/10 wt.% GNP; (**c**,**d**) PLA/10 wt.% GNP compression-moulded composites (the yellow arrows indicate the presence of GNP).

Before observing the filler localisation in the PLA/PCL/GNP composites, the morphology of the PLA/GNP and PCL/GNP composites was investigated using SEM. In Figure 2, PCL/10 wt.% GNP (Figure 2a,b) shows different orientation of the graphene aggregates (indicated by yellow arrows) as compared to the PLA/10 wt.% GNP composite (Figure 2c,d). The graphene orientations in each of these samples seem to be perpendicular to each other.

**Figure 3:**
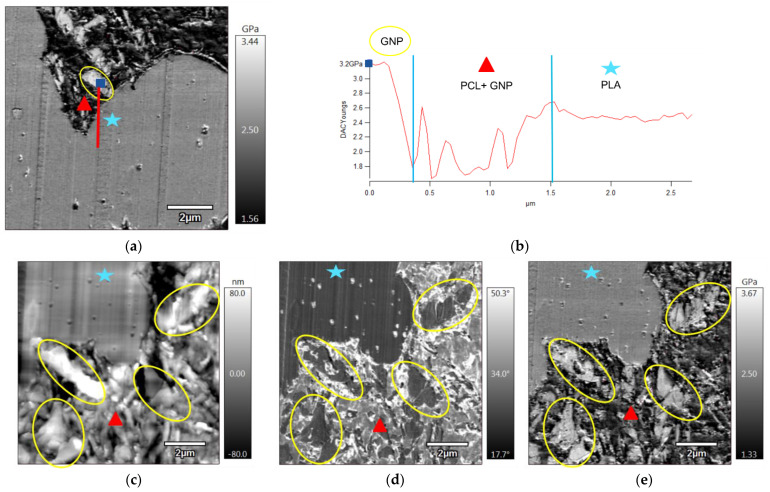
AFM images of PLA60/PCL40/GNP, where (**b**) shows the components of the profile drawn starting from the blue square on (**a**); (**c**–**e**) represent the topography, phase, and elastic modulus images of another observed zone on the same surface (the yellow ellipses surround the GNP, the blue stars denote the PLA phase, and the red triangles indicate the graphene containing PCL phase).

Figure 3b shows the different components along an AFM profile (indicated by a red line) drawn in Figure 3a for mapping the elastic modulus. Due to the differences in stiffness of the different composite components, it was possible to identify the polymer phases and the preferential localisation of GNP in PLA60/PCL40/10 wt.% GNP composite. First, however, for the interpretation of this profile, elastic modulus of each of the three composite constituents should be known. In this case, Young’s modulus of PLA is 3500 GPa [60], that of PCL is 560 ± 50 MPa [61], and that of graphene fluctuates between 0.98 and 1.04 TPa [62]. Then, starting from the blue point in Figure 3a,b, the highest modulus peak is ascribed to a GNP particle (surrounded by a yellow ellipse in Figure 3a). Subsequently, the red triangle in both Figure 3a,b indicates the presence of PCL filled with 10 wt% GNP particles. Fluctuations of stiffness are observed indicating the presence of particles in the PCL phase. Finally, the blue star denotes the PLA phase having an intermediate modulus between the GNP and PCL. The stability of the modulus profile (Figure 3b) of the PLA phase emphasises its purity and emptiness from any GNP particles. 

Further AFM images were taken on another zone of the sample’s surface. Observations are shown in Figure 3c (topography), d (phase), and e (elastic modulus). Considering the latter, the brighter zone which possesses the greatest elastic modulus indicates the GNP (surrounded by yellow ellipses), the darkest zone which has the lowest elastic modulus (indicated by the red triangle) shows the PCL phase, and the grey zone with intermediate elastic modulus between the GNP and PCL indicates the PLA presence (indicated by the blue star). The topography (Figure 3c) and the phase (Figure 3d) images give the same information where in the topography image the greatest roughness (brightest colour) is due to the GNP presence, and in the phase image, the highest angle (brightest colour) indicates generally the softest material, which is PCL.

**Figure 4:**
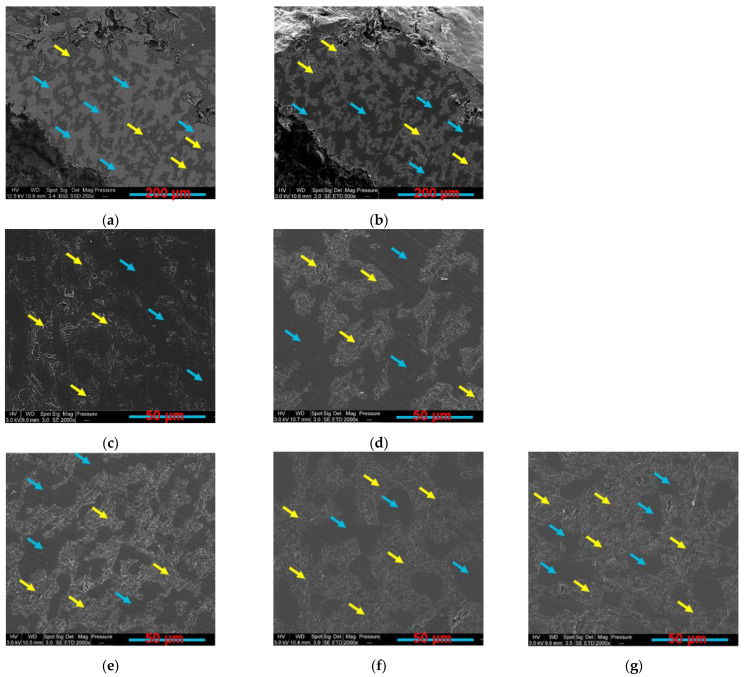
SEM images of PLA60/PCL40/GNP, (**a**) BSE and (**b**) SE and SE images at lower magnification: (**c**) PLA65/PCL35/GNP; (**d**) PLA60/PCL40/GNP; (**e**) PLA50/PCL50/GNP; (**f**) PLA40/PCL60/GNP; (**g**) PLA30/PCL70/GNP compression-moulded composites (the yellow arrows indicate the GNP rich PCL phase and the blue ones indicate the PLA phase).

Other PLA/PCL/GNP compositions also show a selective localisation of GNP in the PCL phase. Figure 4 shows a series of samples containing 10 wt.% of GNP and PLA content, ranging between 65 wt.% (Figure 4c) to 30 wt.% (Figure 4g). In these images, GNP are totally localised in the PCL phase (indicated by yellow arrows) with a total absence in the PLA phase (indicated by blue arrows). The phases were identified in these secondary electron images by comparing a BSE image (Figure 4a) with a SE image (Figure 4b) of PLA60/PCL40/GNP. The brighter phase in the BSE image is the oxygen content richer polymer phase (PLA phase), as indicated by blue arrows, whereas it is the opposite for the SE image (brighter phase is the PCL phase as indicated by yellow arrows). 

The selective localisation of nanofillers in a specific phase or at the interface of immiscible polymer blends is mainly controlled by the combined action of the thermodynamics and the kinetics. To reduce the free energy of the system, nanoparticles tend to selectively locate in the more thermodynamically favourable phase during the melt mixing process. The thermodynamic preference of nanoparticles could be determined by the wetting coefficient, which measures the tendency of a liquid phase to spread [8]. It can be calculated from the ratio of the interfacial tensions (the subtraction of the interfacial tensions between the polymer/filler systems over the interfacial tension of the polymer/polymer system). In this framework, based on the contact angle measurements and the wetting coefficient calculations that were carried out in our previous work [55], GNP should have preference to the PLA phase. However, these observations do not consider the process temperature and what happens during high shear melt blending at 180 °C. 

From a kinetic point of view, nanoparticles tend to localise in the phase with lower viscosity during melt mixing, since the less viscous polymer wets nanoparticles more easily in the early stages of melt mixing [8]. In this work, the complex viscosity of PLA (819.47 ± 114.06 Pa·s at 115 rad·s^−1^) is greater than that of PCL (183.67 ± 15.94 Pa·s at 115 rad·s^−1^), so the nanoparticles can easily migrate into the PCL phase during melt mixing. Several previous works highlighted the same observation of GNP preferential positioning in the PCL phase rather than the PLA phase [55,63,64]. 

### 3.2. Theoretical Prediction of the Co-Continuity

The continuous phase depends mainly on the composition and the viscosity ratio of the components. For instance, by starting with polymer A and consequently adding to it polymer B, the dispersion of polymer B in A is obtained. If the volume fraction of polymer B still increases, the phase inversion point is reached, after which polymer B becomes the continuous phase and polymer A is the dispersed phase [65]. In other words, increasing the dispersed volume fraction at constant melt blending speed may result first in a co-continuous system and eventually a phase inversion point, beyond which the reversed system is obtained. 

Several empirical and semi-empirical models have been developed over the last three decades to estimate the phase inversion composition in terms of processing conditions and material properties. Paul and Barlow proposed one of the simplest models that uses the viscosity ratio of the two polymer phases to estimate the phase inversion point. According to these authors, the less viscous phase will have the greater ability to be the continuous phase [66]. In addition, Ho et al. [67], Kitayama et al. [68], and Everaert et al. [69] have modified the general equation by introducing a prefactor and/or an exponent to better fit the experimental results. Instead of using the viscoelastic parameters, Metelkin and Blekht [70] were the first to introduce the empirical relationship to predict the phase inversion using the concept of capillary instabilities of individual layers. Utracki [71] developed another approach based on the suspension theory using the intrinsic viscosities [η] and maximum packing volume fraction ϕ_m_. Steinmann et al. [38], proposed an approach based on the assumption that the shape relaxation times of blend components meet a maximum at the phase inversion point. Bourry and Favis [33] have developed a different approach using the storage modulus instead of the viscosity ratios by considering the elastic contribution of the blend components.

In most of the above-mentioned models, the volume percentages of PLA and PCL at the phase inversion point can be calculated by using the complex viscosities of both polymers, owing to the influence of the viscosity ratio on the morphology of the polymer blends [6]. As mentioned before, graphene is completely localised in the PCL phase, thus the rheological parameters (complex viscosity and storage modulus) of pure PLA and PCL/GNP phases were taken into consideration in the different models. Yet, only the values at 120 rad·s^−1^ (close to the shear rate existing during twin-screw extrusion by process eleven mini extruder) and 628 rad·s^−1^ (Table 2) were used in the calculations due to the fitting of only these results with the various models. Table 3 includes the equations and the results of each model at the two different shear rates which show that the inversion point was not affected by the shear rate variation due to the close results. In the following sections, the combination of various results will indicate the sample which has the best co-continuity, and therefore, it will be considered as the experimental phase inversion point and will be compared with the results of the different models in this section.

### 3.3. Experimental Prediction of the Co-Continuity Range in the Compression-Moulded Samples

#### 3.3.1. SEM Results

**Figure 5:**
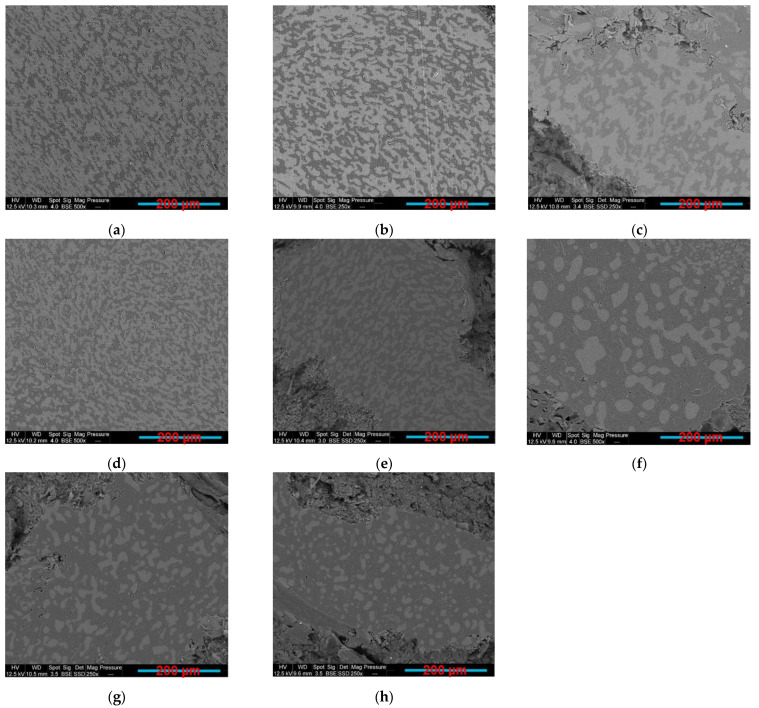
SEM BSE images: (**a**) PLA70/PCL30/GNP; (**b**) PLA65/PCL35/GNP; (**c**) PCL60/PCL40/GNP; (**d**) PLA55/PCL45/GNP; (**e**) PLA50/PCL50/GNP; (**f**) PLA45/PCL55/GNP; (**g**) PLA40/PCL60/GNP; (**h**) PLA30/PCL70/GNP (PLA is the white phase and PCL is the grey phase).

As the composites with co-continuous morphology may offer more interesting combination of properties, such as the electrical ones as compared to blends with dispersed structure, there has been a growing interest in this type of materials. In this work, samples having PLA content between 70 wt.% and 55 wt.% (Figure 5a–d) were found to be co-continuous. This co-continuity was lost at lower PLA percentage (50–30 wt.%) (Figure 5e–h), where the nodules of PLA are dispersed in the continuous GNP-rich PCL phase. It was long ago believed that the co-continuous morphologies exist only close to the phase inversion point, but recently several researchers showed that a range of co-continuity can exist, rather than a single point. The co-continuous morphologies can be formed at the initial, intermediate, and final stages of melt blending depending on several factors, such as the mixing conditions, the type of extruders, etc. [74]. 

There is a debate in the literature regarding the mechanism of formation of co-continuity in polymer blends, whether it starts by droplet deformation/breakup or by a sheet forming mechanism [46]. In unfilled polymer blends, the thermodynamic stability of the elongated domains depends on the polymer–polymer interfacial tension that causes the retraction of the minor phase to a spherical shape or its fragmentation into small droplets. The addition of nanoparticles affects the interfacial tension and the rheological behaviour, and consequently, the co-continuity equilibrium [75,76,77]. Indeed, there is no clear understanding of the mechanisms that lead to the formation of the co-continuity in the presence of nanofillers, even if it has been reported that the rheology contributes greatly in this aspect. When the nanoparticles are selectively located in one polymer phase, the increase of the elasticity and viscosity of the host slows down its shape relaxation and breakdown processes to stabilise the irregularly shaped domains. This may eventually result in stable co-continuous morphologies [58]. 

Several authors have obtained co-continuous blends with the selective localisation of the nanoparticle in one polymer phase. For example, Filippone et al. [45] found that PA6/PP/clay systems, in which the clay is selectively localised in the PA6 minor phase, exhibited a co-continuous microstructure. To explain their results, they proposed a gelation mechanism, where the PA6 chains coat the organoclay network. Li et al. [78] also suggested a mechanism for the formation of a co-continuous structure in clay filled PA6/(poly(phenylene oxide) (PPO) blends. They indicated that the filler presence in the PA6 phase refines the domain size and increases the effective filling volume of the minor phase. The enhanced droplet/droplet interactions finally lead to the formation of the co-continuous structure. Cui et al. [79] have obtained the double electrical percolation in immiscible PP/Novolac blends filled with CB particles that were selectively localised in the thermosetting Novolac phase. At a processing temperature of 190 °C, Novolac has a lower melt viscosity than PP, and this favours the CB incorporation into the Novolac resin. This preferential localisation increased the Novolac viscosity until it became equivalent to PP, thus facilitating the co-continuity formation. According to these authors, it is indispensable for a co-continuous morphology formation that the shear stresses within a system are greater than the interfacial stress in order to favour the distributive mixing and thus the phase elongation. The greater interfacial stress between the blend polymers results in smaller dispersive mixing. Elongated particles are not stable in such flow fields, resulting in a dispersed phase morphology. 

#### 3.3.2. Electrical Resistivity and Solvent Extraction Method Results

The electrical conductivity of composites is very sensitive to their microstructure. Moreover, in order to obtain electrically conductive composites, the electrons tunnelling (filler particles are close but not in contact) and/or direct contact effects should exist [59]. In this context, composite nanomaterials containing a 10 wt.% of GNP and a PLA concentration varying between 0 and 100 wt.% were fabricated and tested. The electrical volume resistivity results, the electric current at 5 v, and the LED photographs at 5 v are presented in Table 4. The LED photographs were taken after the assembly of a circuit consisting of a LED, a voltage generator (5 v), and the sample in series. The lowest electrical resistivity results are for samples containing PLA content, varying between 55 and 70 wt.% (greater electric current and LED luminosity as compared to the other samples). This can be attributed to the double electrical percolation phenomenon that exists in these samples (proved by the full continuity of the phases in the following section using the solvent extraction method).

**Figure 6:**
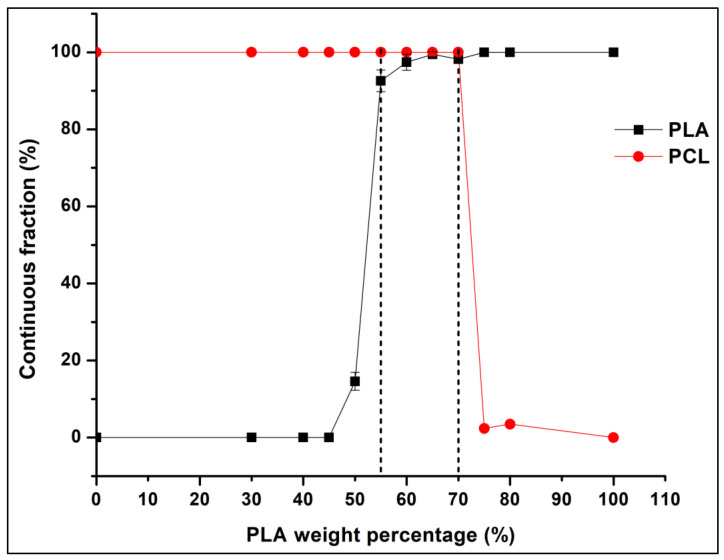
Continuous fraction percentage plot of PLA and PCL phases of each compression moulded composite containing 10 wt.% of GNP versus the PLA weight percentage (these results were obtained from the solvent extraction method).

Both the microstructural and electrical resistivity results led to the same conclusion regarding the composites co-continuity range, which is between 55 wt.% and 70 wt.% of the PLA. The solvent extraction method was implemented to quantify the 3D co-continuity by extracting the PCL phase using toluene solvent. Figure 6 exhibits the plot of the continuous fraction percentage of each polymer as a function of the PLA weight percentage in the 10 wt.% GNP compression-moulded composites. The PLA curve has a classical sigmoid shape. At a low percentage of PLA (≤45 wt.%), its continuity is zero. This means that the PLA phase is dispersed as droplets in the PCL phase (as observed in Figure 5f–h). At 50 wt.% of PLA, the beginning of the coalescence of PLA droplets starts (also obvious in Figure 5e) and continues until 55 wt.%, which corresponds to the onset of the co-continuity. Starting from this value, the PLA phase becomes almost fully continuous. The co-continuity interval extends from 55 wt.% to 70 wt.% of the PLA (the last PLA percentage at which both polymers are fully continuous) (exhibited in Figure 5a–d). This range is between the dotted lines shown in Figure 6 and was determined in the same way as a reference [80]. The PCL curve has the same shape, but it has broader full continuity range (0–70 wt.% of PLA) as compared to PLA due to its lower complex viscosity. The dispersed PCL structure in the PLA phase is observed only beyond 70 wt.% of the PLA (almost zero continuity of the PCL phase). These results emphasise the co-continuity range already observed by the microstructural and electrical analyses.

What can also be realised in Table 4 is that as the percentage of PCL increases from 35 wt.% to 100 wt.%, the electrical resistivity increases because of the lower GNP confinement and their greater dispersion in the larger PCL phase. Meanwhile, PLA80/PCL20/GNP has high electrical volume resistivity (4900 ± 90.19 Ω·cm), although it has the lowest PCL content in this sample. The morphology of this sample demonstrated in our previous work [55] can explain this result. The scarcity of the graphene-filled PCL content that exists in a nodular form in the continuous PLA matrix causes the separation of the graphene clusters by the insulating PLA chains. In addition, comparing the PLA/GNP results (4865.9 ± 65.11 Ω·cm) with the PCL/GNP ones (500 ± 30.33 Ω·cm) shows a large difference among them (ratio of 9.7). This is due to the different GNP orientation in the PCL phase in comparison with the PLA matrix (Figure 2). In the PCL/GNP composite, graphene flakes are along the direction of the copper electrodes (of the electrical resistance measurement setup sample holder) (Figure 2a,b), which makes the transfer of the electrons easier as compared to the orientation of the GNP perpendicular to the copper electrodes direction in the PLA matrix (in PLA/GNP composite, Figure 2c,d). The four-probes electrical resistance measurement setup is demonstrated in our previous work [55].

#### 3.3.3. Melt Shear Rheology Results

**Figure 7:**
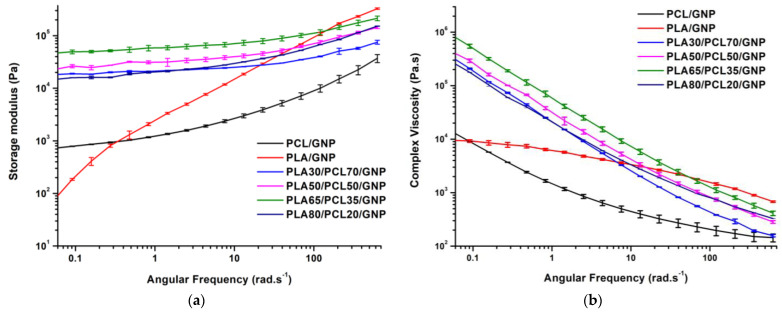
(**a**) Storage modulus; (**b**) complex viscosity versus angular frequency of compression-moulded composites containing 10 wt.% of GNP.

The rheological properties of the polymer nanocomposites are sensitive to their inner microstructures. Thus, the storage modulus and complex viscosity curves, obtained after performing melt shear rheology tests at 180 °C, are depicted in Figure 7a,b, respectively. Conversely to PLA/GNP, the complex viscosity and storage modulus curve shape of PCL/GNP is similar to that of the ternary composites. This confirms that all the nanocomposites, except the PLA/GNP, exhibit a yield stress behaviour that is not exclusive for the co-continuous composites [58,81].

In the low frequency region, the comparison between the storage modulus curves gives the following order: PLA65/PCL35/GNP > PLA50/PCL50/GNP > PLA30/PCL70/GNP > PLA80/PCL20/GNP. For all these systems, the storage modulus at low frequency follows the same trend as that of the electrical volume conductivity for the same structural reasons. This result was obtained in a previous work, performed by Hadaeghnia et al. [81], who explained the increase of low frequency storage modulus by the formation of a strong percolated structure of graphene particles and the presence of a co-continuous structure in PA6/PEO blends. Lyu et al. [82] found superior storage modulus of the co-continuous poly vinyl chloride (PVC)/PLA blends as compared to the composites with dispersed morphology because of the better interaction stresses between the two polymer phases that hindered the chain relaxation and enhanced the elasticity. 

Concerning the composites of this study, the PLA and PCL contents are not constant. Thus, in principle, the sample that contains more PLA content should exhibit higher storage modulus because of the greater storage modulus of pure PLA (55,861 ± 1121.31 Pa at 115 rad·s^−1^ which corresponds approximately to the shear rate level during twin-screw extrusion processing) as compared to pure PCL (3678 ± 398.26 Pa at 115 rad·s^−1^) [55]. Yet, PLA80/PCL20/GNP, although it contains the greatest PLA content, it shows the lowest storage modulus as compared to the other ternary composites. The reason might be the absence of the strong interconnected graphene network in this sample as indicated by the high electrical volume resistivity (4900.43 ± 90.19 Ω·cm). 

Moreover, the formation of a plateau in the low frequency region is a clue regarding the presence of a strong particulate network [83,84,85,86]. In addition, because PLA65/PCL35/GNP composite demonstrates this plateau, this emphasises the fact that graphene is more concentrated in the PCL phase in this sample [59]. The continuous graphene network restricted the movement of the PCL chains and the PLA chains at the interface [87], which caused the increase of the complex viscosity of the co-continuous sample at elevated temperature (Figure 7b). In conclusion, the selective localisation of the nanofiller, along with the co-continuous structure, are the reason behind the superior viscous and elastic properties of PLA65/PCL35/GNP composite [88]. 

Thus, by combining the SEM, electrical volume resistivity, solvent extraction, and rheological conclusions, PLA65/PCL35/GNP shows the best properties that are linked to the more interesting co-continuous structure of this sample. When referring to the models demonstrated in Table 2, the Kitayama and Bourry–Favis models, which predict that PLA58/PCL48/GNP and PLA60/PCL40/GNP are the phase inversion points, respectively, at 628 rad·s^−1^, are the closest to our experimental findings. 

#### 3.3.4. DSC Results

**Figure 8:**
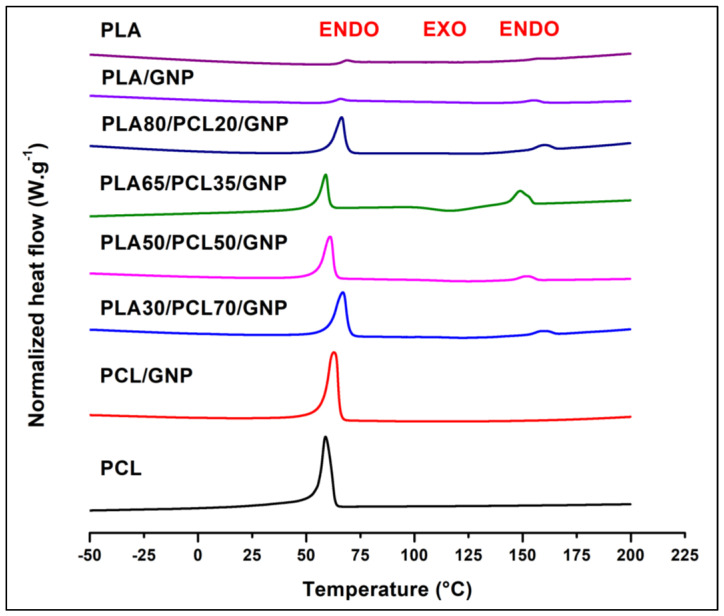
DSC second heating step curves of PLA, PCL, and their binary and ternary composites.

In an attempt to correlate the co-continuity with the degree of crystallinity of each polymer, DSC tests were performed. The results are demonstrated in Figure 8 and Table 5. The percentage of crystallinity of PLA was improved in the case of the ternary composites as compared to the binary composite and the pure polymer that showed no exothermic peak because of its slow crystallisation [89]. This indicates that the presence of PCL acted as a nucleating agent for the PLA crystallites. This percentage was maximal in the sample that possesses a co-continuous structure (PLA65/PCL35/GNP) (7.213%). As an explanation, the presence of selectively localised GNP at the continuous interface should have promoted the crystallisation of this polymer through a heterogeneous nucleating effect [90,91]. The improvement of the PLA crystallisation was obtained in another nanocomposite blend system that contained selectively localised CNT in the poly butylene succinate (PBS) phase and at the interface between the PLA and PBS [88]. In the present work, the PCL crystallinity percentage was improved only in the case of the co-continuous composite (58.5% as compared to 49.5% of pure PCL). This can be due to the selectively localised dense continuous graphene network present in this phase that promoted its fractionated crystallisation [91]. It has been mentioned that the melt crystallisation of the PCL component in the PLA/PCL blend composites can be strongly influenced by the carbon nanofiller selective localisation which can trigger an evident nucleating effect. This can be the result of three roles played by the nanofiller: a heterogeneous substrate to facilitate nucleation, a physical barrier to retard the spherulite growth [90], and an inhibitor of the polymer chains mobility to increase their incorporation into the growing crystals [92]. Several studies have already highlighted that nucleation caused by nanoparticles is highly dependent on the quality of their dispersion and the surface area, which in turn modifies the number of nucleation sites available to start the process [93,94]. 

#### 3.3.5. TGA Results

**Figure 9:**
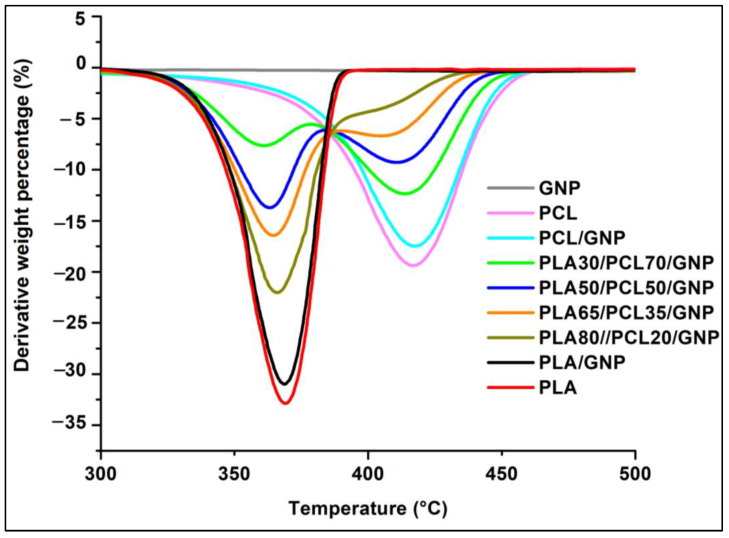
TGA curves of PLA, PCL, and their binary and ternary composites.

To evaluate the thermal stability of the nanocomposites, thermogravimetric analyses were carried out under nitrogen atmosphere, and the results are presented in Figure 9 and Table 6. Pure PCL shows greater thermal stability as compared to pure PLA, due to its superior maximal degradation temperature (greater by 50 °C). Both polymers show a single degradation step. Thus, their blend composites showed a double-staged degradation that corresponds to the individual degradation of the PLA and PCL components. The thermal stability was not improved in the case of the composites as compared to the pure polymers due to the similar values of the onset and maximal degradation temperatures. When comparing the theoretical and experimental char yield at 600 °C, it can be noticed that there is char formation improvement only in the case of the PLA65/PCL35/GNP, which can be attributed to the dense graphene network that protected the PCL phase, inhibited its mobility, and acted as a barrier to the mass transfer during thermal degradation [88,95]. The lowest char yield was obtained for the PLA/GNP composite (lower than the theoretical char yield). This can be probably attributed to the GNP aggregates present in this sample (Figure 2) that played no role in protecting the PLA chains against the thermal degradation.

All the previous results led to a conclusion that PLA65/PCL35/GNP is the best formulation in terms of microstructure and properties. As a result, PLA65/PCL35 composition was chosen for the following three various studies:

1. Influence of the annealing time on the co-continuity and therefore on the electrical conductivity;
2. Influence of the twin-screw extrusion protocol on the co-continuity and the electrical conductivity;
3. Effect of the graphene content on the co-continuity and the electrical conductivity.

### 3.4. Influence of Annealing Time during Compression Moulding on the Co-Continuity

**Figure 10:**
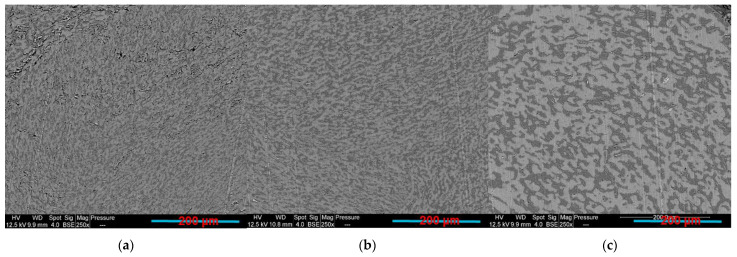
PLA65/PCL35/GNP compression-moulded composite during: (**a**) 10 min; (**b**) 20 min; (**c**) 35 min.

The increase of the compression moulding duration effect on the PLA65/PCL35/10 wt.% GNP composite co-continuous morphology was studied. Figure 10 shows that the increase of compression moulding time from 10 min (Figure 10a) to 35 min (Figure 10c) caused the increase of the size of both phases. The annealing effect, which is more pronounced with the longer duration of thermo-compression, is the reason behind this morphological evolution. For double percolated structures, thermal annealing can induce phase coarsening which can effectively improve the phase continuity of the co-continuous structure and reduce the interfacial area [96]. The coarsening of immiscible co-continuous polystyrene (PS)/poly (methyl methacrylate) (PMMA) blends during quiescent annealing has already been studied in a previous work [52]. The volume average pore diameter was used to characterise the coarsening effect which was evidenced by the pore size growth. The mercury intrusion porosimetry (MIP) was used to estimate this diameter after the selective extraction of each polymer phase using a convenient solvent. Consequently, a direct relationship between the pore size (R) and the annealing time (t) was deduced (R∼kt). In another work [97], the electrical properties of PS/PMMA/MWCNT composites with double percolation structures were improved by thermal annealing. The greater coalescence of small phases caused the construction of more a perfect co-continuous structure with greater conductive pathway continuity. Moreover, the re-aggregation of loose MWCNT into a better network in the preferential PMMA phase during the thermal annealing process helped in improving the electrical conductivity. The construction of conductive networks by thermal annealing treatment in polymer matrices was reported in many of works [96,98,99,100,101,102,103,104]. In the present work, the increase of the annealing time also leads to an increase in the electrical conductivity (Table 7). This might be due to the greater time given to GNP to form a better-connected conductive pathway that was facilitated by the greater PLA continuity (increase of PLA continuity percentage with the annealing time, Table 7).

### 3.5. Influence of the Extrusion Protocol

**Figure 11:**
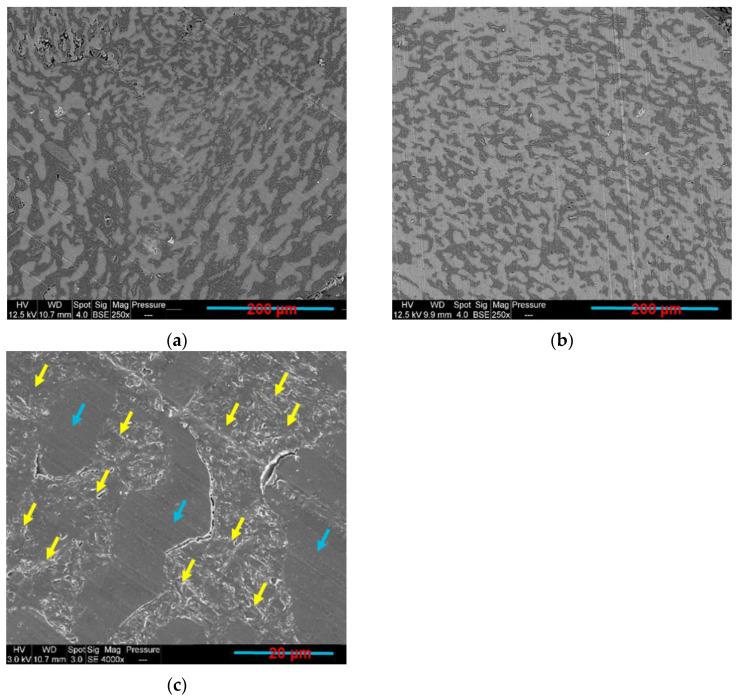
PLA65/PCL35/GNP compression-moulded sample extruded: (**a**,**c**) in two steps; (**b**) in one step (**b**) (the yellow and blue arrows indicate PCL/GNP and PLA phases, respectively).

**Figure 12:**
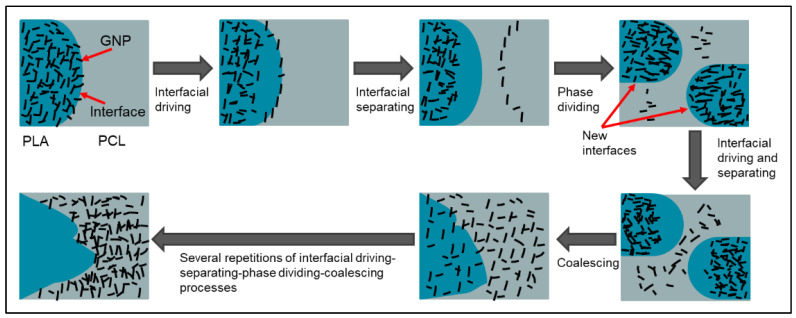
Scheme representing the migration mechanism of GNPs from PLA to PCL during twin-screw extrusion (image reproduced from reference [105]).

It has been reported that the processing sequence is one of the important factors that influences the electrical properties of various binary blend composites [79]. To compare with PLA65/PCL35/10 wt.% GNP composite prepared by one extrusion step, the same composite by two extrusion steps was prepared and evaluated. In the latter, the GNPs were first blended with PLA, then the resulting PLA/GNP pellets were added to the PCL in a second step. The results showed a co-continuous structure regardless of the different preparation procedures (Figure 11a,b). In the two-step extruded composite, the GNP particles are only present in the PCL phase as indicated by the yellow arrows, whereas the PLA phase, marked by the blue arrows, seems GNP-free (Figure 11c). This observation highlights the migration of the GNPs from the PLA phase to the PCL during extrusion. As a consequence, the viscosity of the PCL phase increased to promote the co-continuity of both polymer phases, as explained by Tao et al. [105]. These authors observed the migration phenomenon of carbon nanotubes in co-continuous PLA/PCL blends. The successive phenomena of interfacial driving—separation—division—coalescence are responsible for the nanofiller migration from PLA to PCL, based on their research. Figure 12 shows these phenomena in the PLA/PCL/GNP system of this study. 

Similar PLA continuity percentage, probably favoured by the GNP migration, led to similar electrical resistivity in both composites (Table 8). This can be attributed to the similar concentration of GNPs in the PCL phase. The complete migration of the nanoparticles from one polymer phase to another during the melt blending is not always the case. Cui et al. [79] have obtained a much greater electrical volume resistivity of the (PP+CB)/Novolac composite (PP and CB were melt blended together at first using an internal mixer and later mixed with Novolac) as compared to the simultaneously blended composite. In the latter, the CB particles are selectively localised in the Novolac phase. In the former, because of the greater affinity of the CB particles to the Novolac phase, these particles were migrated from the PP phase but in a partial manner. 

### 3.6. Influence of GNP Content on the Co-Continuity

**Figure 13:**
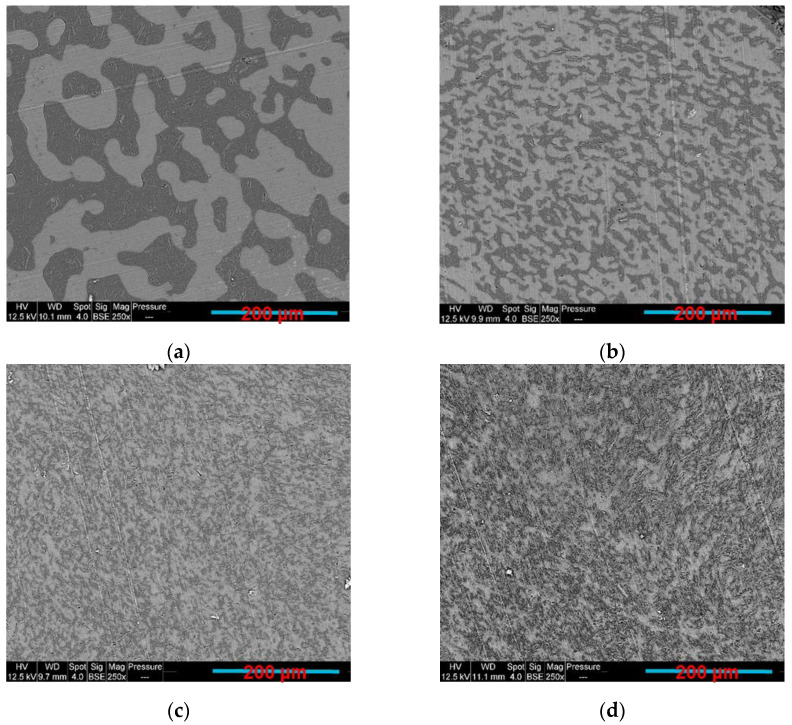
SEM images of compression-moulded PLA65/PCL35 blend composites reinforced with: (**a**) 5 wt.% GNP; (**b**) 10 wt.% GNP; (**c**) 15 wt.% GNP; (**d**) 20 wt.% GNP (white phase is PLA and grey phase is PCL).

**Figure 14:**
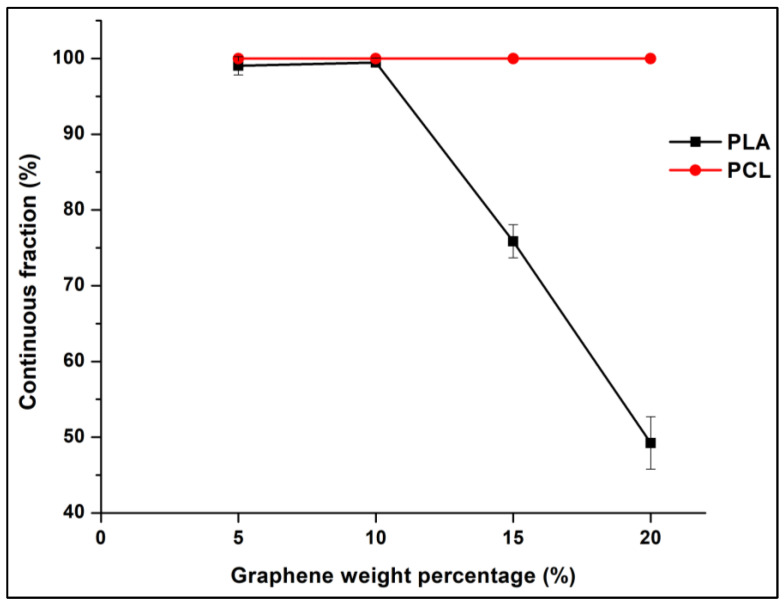
Variation of the continuity percentage of the PLA and PCL fractions with the increase of graphene percentage in PLA65/PCL35/GNP composites.

For the objective of verifying whether the variation in the graphene content would maintain the co-continuity, GNP percentage was varied from 5 wt.% to 20 wt.% in the PLA65/PCL35 blend composites. Figure 13 and Figure 14 present the SEM observations of the different samples and their continuity fraction percentages, respectively. Combining the results of both figures shows that the co-continuous morphology was maintained when the graphene percentage was equal to and less than 10 wt.%. Beyond this percentage, the PLA continuity percentage decreased to approximately 50% at 20 wt.% of graphene. The increase of the viscosity of the PCL and the consecutive shearing entailed a reduction in the PLA domain’s size to therefore perturb their continuity. Consequently, the barrier to the coalescence of the PLA phase was increased. Although the presence of graphene nanoparticles inside the minor phase increases the elasticity and therefore could stabilise the elongated domains, a threshold of this stabilisation could exist. In the PA6/PEO system reinforced by graphene, the co-continuity was reduced with the increase of the graphene content due to the reduction of coalescence [81]. Additionally, it was the case in PA6/ABS system, where with the increase of MWCNT content, the co-continuous structure is more refined [21]. Table 9 demonstrates the electrical volume resistivity of our composites. The increase of the graphene percentage resulted in a denser GNP network formation, and therefore the electrical volume resistivity decreased to 2 × 10^−3^ Ω·cm at 20 wt.% of GNP. In conclusion, although it has greater resistivity, PLA65/PCL35/10 wt.% GNP composite represents a compromise between robust co-continuity and good electrical conductivity. 

### 3.7. Influence of 3D Printing on the Co-Continuity

**Figure 15:**
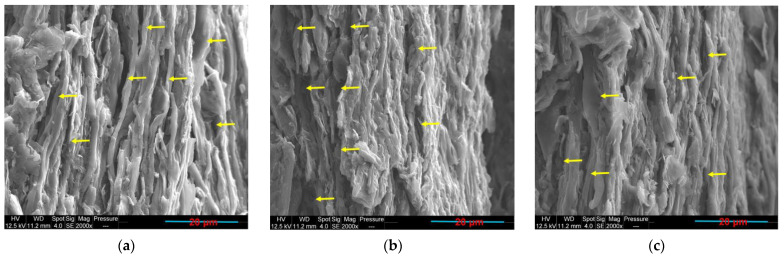
SEM images of surface etched 3D-printed PLA65/PCL35/GNP composite, (**a**–**c**) correspond to different parts of the surface (the yellow arrows point to the pores where PCL elongated nodules were present) (the 3D-printed filaments orientation is the same as the orientation of the PCL pores).

**Figure 16:**
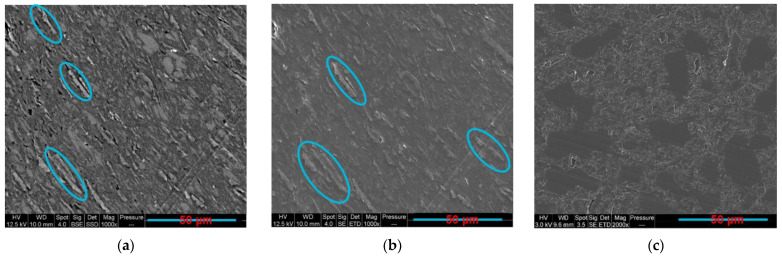
The SEM images: (**a**,**b**) PLA30/PCL70/GNP 3D printed; (**c**) compression-moulded composites (the brighter phase is PLA in Figure 15a and the blue ellipses surround the graphene aggregates) (the 3D-printed filaments have the same orientation as the GNP aggregates).

The co-continuous structure can help in increasing the printing performance and the possibility to produce conductive composites at low percolation thresholds [31,82,106]. Thus, several composites containing 10 wt.% of GNP and different proportions of PLA and PCL were prepared through fused filament fabrication (FFF). The electrical results of these samples, along with the results of the compression-moulded samples, are demonstrated in Table 10. The objective of these experiments is to investigate the possible conservation of the co-continuous structure after the additive manufacturing process, and therefore, to see the consequences on the electrical resistivity. The morphology of toluene etched 3D-printed PLA65/PCL35/10 wt.% GNP is demonstrated in Figure 15. The distinction between the PLA and the PCL phases was not possible for the 3D-printed samples with high PLA content, therefore the surface etching technique was implemented prior to SEM. The presence of elongated pores that correspond to the absent PCL phase indicates that this sample is co-continuous. This can be confirmed by its lowest electrical resistivity, which was able to illuminate the LED, as compared to the other printed samples. In addition, the co-continuous compression-moulded sample shows lower electrical volume resistivity. This can be due to the presence of partial co-continuity that starts and ends in each deposited filament and that is not extended all over the volume as it is the case in the compression-moulded samples. The high shear rate (approximately 125 s^−1^) at the nozzle may also have an impact on the microstructure. Moreover, oriented GNP aggregates in the 3D-printed sample can be another reason. Figure 16a,b show some oriented graphene aggregates, surrounded by blue ellipses, in PLA30/PCL70/GNP 3D-printed composite possessing a sea-island morphology. These aggregates were absent in the compression-moulded sample (Figure 16c) that shows good and random dispersion of graphene in the PCL phase. These microstructural differences are the reason behind the inferior electrical resistivity in all the compression-moulded samples as compared to the printed ones. It is noteworthy that as the percentage of PCL increases from 35 wt.% to 70 wt.%, the electrical volume resistivity increases. The same trend was also observed for the compression-moulded samples (Table 10).

## 4. Conclusions

This study has shown for the first time the achievement of a double electrical percolation phenomenon in PLA/PCL/GNP nanocomposites. The influence of different processes on this phenomenon has been scrutinised. The weight percentage of graphene was initially fixed at 10 wt.%, and the respective percentages of PLA and PCL were varied between 0 and 100 wt.%. The AFM and the SEM techniques showed a selective localisation of GNPs in the PCL phase, and the co-continuity range was from PLA55/PCL45/GNP to PLA70/PCL30/GNP in the compression-moulded samples as it was confirmed by the solvent extraction experiments. The electrical volume resistivity was the lowest in this range with the best results for the PLA65/PCL35/GNP. The melt shear rheological parameters, such as the storage modulus and complex viscosity, were the greatest for the PLA65/PCL35/GNP at the low angular frequency indicating the presence of the densest graphene-percolated structure. The thermal analysis using TGA and DSC showed that PLA65/PCL35/GNP has the greater char formation and percentage of crystallinity of both polymers. Various viscosity and storage modulus-based models were applied to predict the phase inversion point that represents the maximum of the co-continuity. Accordingly, the Kitayama and Bourry–Favis models were the closest to our experimental findings since they predict at 628 rad·s^−1^ that PLA58/PCL42/GNP and PLA60/PCL40/GNP are the phase inversion points, respectively, and we have obtained that PLA65/PCL35/GNP has the greatest co-continuity. Moreover, the influence of various processing parameters on the co-continuity was studied. As expected, the size of the co-continuous domains increased due to the annealing effect, which led to the resistivity reduction. All the samples exhibited a co-continuous structure, starting from 10 min of compression moulding with an increase in PLA continuity percentage with the compression moulding time. The sample fabricated in the two extrusion steps showed a phenomenon of complete migration of GNPs from the PLA phase to the PCL phase with a co-continuous microstructure establishment. Furthermore, the increase of the graphene percentage led to a decrease in the polymers’ network size and co-continuity due to the variation of the complex viscosity ratio of the two polymers and the barrier effect to coalescence. The 3D-printed PLA65/PCL35/GNP composite showed the best electrical conductivity results due to partial co-continuous structure, conversely to the other compositions. Yet, the compression-moulded samples still have superior electrical conductivity due to their co-continuous microstructure which is extended all over the surface. This is not the case for the 3D-printed sample whose co-continuity size is restricted within the diameter of each deposited filament. Overall, the results demonstrate that the investigated PLA/PCL/GNP composites are promising materials for technological applications, such as chemical sensors, flexible electronic devices, electrical circuit printing, and electromagnetic interference shielding (EMI).

## Figures and Tables

**Table 1:** The three main series of extruded composites and the objective behind preparing each composition.

Extruded Bionanocomposites	Graphene Percentage	PLA and PCL Percentages	Objective
PLAx/PCLy/10 wt.% GNP	10 wt.% GNP	x and y range between 0 and 100 wt.% (with respect to the total weight fraction of 90 wt.% of polymers),	Exploring the co-continuity range in the compression-moulded samples at 10 wt.% of GNP,
PLA65/PCL35/x wt.% GNP	x varies between 5 and 20 wt.%	Fixed at 65 wt.% and 35 wt.% for PLA and PCL, respectively, (with respect to the total weight fraction of 90 wt.% of polymers),	Exploring the influence of the graphene percentage on the co-continuous microstructure in the compression-moulded samples,
PLA65/PCL35/10 wt.% GNP (2 extrusion steps)	10 wt.%	PLA and 10 wt.% of GNP (with respect to the PLA/PCL/GNP composite) were blended and subsequently the resulting composite was melt-blended with PCL (PLA and PCL proportions were 65 and 35 wt.% with respect to the total weight of the 90 wt.% of polymers).	Studying if mixing GNP with the less favourable PLA phase would cause its migration to the more favourable PCL phase and the consequences of this migration on the co-continuous microstructure,

**Table 2:** Complex viscosity and storage modulus results of PLA and PCL/GNP composites at 180 °C and 120 rad·s^−1^ and 628 rad·s^−1^.

Sample Name	Complex Viscosity (Pa·s) (120 Rad·s^−1^)	Complex Viscosity (Pa·s) (628 Rad·s^−1^)	Storage Modulus (Pa) (120 Rad·s^−1^)	Storage Modulus (Pa) (628 Rad·s^−1^)
PLA	1409	545.16	117,630	287,750
PCL/10% GNP	197	144.07	10,168	36,269
PCL/15% GNP	672	280.76	67,115	127,125
PCL/20% GNP	1971	623.83	222,643	329,913

**Table 3:** Different phase inversion models and their results in PLA/PCL/10 wt.% GNP composites at 180 °C and 120 rad·s^−1^ and 628 rad·s^−1^.

Authors	Equation	Results (628 Rad·s^−1^)	Results (120 Rad·s^−1^)	Reference
Paul–Barlow	$\frac{\phi_{\mathrm{PCL}\;+\;M5}}{\phi_{\mathrm{PLA}}}=\frac{\eta_{\mathrm{PCL}\;+\;M5}}{\eta_{\mathrm{PLA}}}$	PLA52/PCL48/10 wt.% GNP	PLA45/PCL55/10 wt.% GNP	[66]
Kitayama et al.	$\frac{\phi_{\mathrm{PCL}\;+\;M5}}{\phi_{\mathrm{PLA}}}=0.887{\left(\frac{\eta_{\mathrm{PCL}\;+\;M5}}{\eta_{\mathrm{PLA}}}\right)}^{0.29}$	PLA58/PCL42/10 wt.% GNP	PLA55/PCL45/10 wt.% GNP	[68]
Steinmann et al.	$\phi_{\mathrm{PLA}}=-0.12\log\left(\frac{\eta_{\mathrm{PCL}\;+\;M5}}{\eta_{\mathrm{PLA}}}\right)\;+\;0.48$	PLA52/PCL48/10 wt.% GNP	PLA51/PCL49/10 wt.% GNP	[38]
Ho et al.	$\frac{\phi_{\mathrm{PCL}\;+\;M5}}{\phi_{\mathrm{PLA}}}=1.22{\left(\frac{\eta_{\mathrm{PCL}\;+\;M5}}{\eta_{\mathrm{PLA}}}\right)}^{0.29}$	PLA49/PCL51/10 wt.% GNP	PLA47/PCL53/10 wt.% GNP	[67]
Everaert et al.	$\frac{\phi_{\mathrm{PCL}\;+\;M5}}{\phi_{\mathrm{PLA}}}={\left(\frac{\eta_{\mathrm{PCL}\;+\;M5}}{\eta_{\mathrm{PLA}}}\right)}^{0.3}$	PLA54/PCL46/10 wt.% GNP	PLA53/PCL47/10 wt.% GNP	[69]
Metelkin–Blekht	$\phi_{\mathrm{PLA}}={\left[1\;+\;\frac{\eta_{\mathrm{PCL}\;+\;M5}}{\eta_{\mathrm{PLA}}}\left[1\;+\;2.25\log\left(\frac{\eta_{\mathrm{PCL}\;+\;M5}}{\eta_{\mathrm{PLA}}}\right)\;+1.81{\left(\log\left(\frac{\eta_{\mathrm{PCL}\;+\;M5}}{\eta_{\mathrm{PLA}}}\right)\right)}^{2}\right]\right]}^{-1}$	PLA47/PCL53/10 wt.% GNP	-	[70]
Utracki	$\phi_{\mathrm{PLA}}=\frac{\left(1\;-\;\log\left(\frac{\eta_{\mathrm{PCL}\;+\;M5}}{\eta_{\mathrm{PLA}}}\right)/(\left[\eta \right].\phi_{m})\right)}{2}$ $\left[\eta \right].\phi_{m}=1.9$ [46,72,73]	PLA53/PCL47/10 wt.% GNP	PLA51/PCL49/10 wt.% GNP	[71]
Bourry–Favis	$\frac{\phi_{\mathrm{PLA}}}{\phi_{\mathrm{PCL}\;+\;M5}}=\frac{{G\prime }_{\mathrm{PCL}\;+\;M5}}{{G\prime }_{\mathrm{PLA}}}$	PLA60/PCL40/10 wt.% GNP	-	[33]

**Table 4:** Electrical volume resistivity and electric current results with the accompanied LED photos at 5 v of the compression-moulded samples containing 10 wt.% of GNP and PLA content between 0 wt.% to 100 wt.%.

Sample	Electrical Volume Resistivity (Ω·cm)	Electric Current (mA) (5 v)	LED (5 v)
PLA/GNP	4865.9 ± 65.11	10^−6^ ± 7 × 10^−4^	No light
PLA80/PCL20/GNP	4900.43 ± 90.19	10^−6^ ± 4 × 10^−3^	No light
PLA70/PCL30/GNP	30.23 ± 1.17	9 ± 6 × 10^−2^	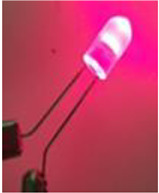
PLA65/PCL35/GNP	25.78 ± 5.11	9.24 ± 4 × 10^−2^	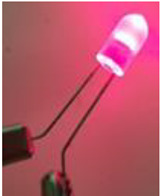
PLA60/PCL40/GNP	53.67 ± 12.24	6.13 ± 8 × 10^−2^	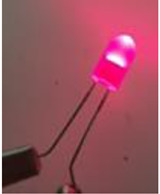
PLA55/PCL45/GNP	66.89 ± 10.26	5 ± 9 × 10^−2^	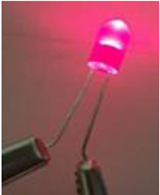
PLA50/PCL50/GNP	75.67 ± 15.19	3.1 ± 5 × 10^−2^	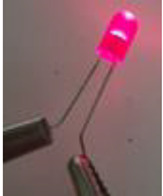
PLA45/PCL55/GNP	104.25 ± 12.38	2.3 ± 10^−2^	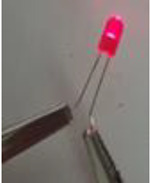
PLA40/PCL60/GNP	125.61 ± 20.81	1.4 ± 2 × 10^−2^	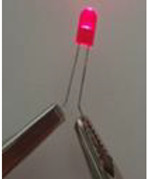
PLA30/PCL70/GNP	237.34 ± 25.33	0.9 ± 10^−2^	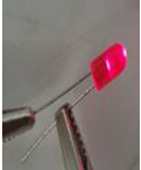
PCL/GNP	500 ± 30.33	0.01 ± 5 × 10^−2^	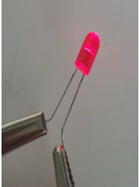

**Table 5:** DSC results of PLA, PCL, and their composites.

Sample Name	T_m_ (PLA) (°C)	T_m_ (PCL) (°C)	T_c_ (PLA) (°C)	X_c_ (PLA) (%)	X_c_ (PCL) (%)
PCL	-	58 ± 0.17	-	-	49.5 ± 0.1
PCL/GNP	-	62 ± 1.7	-	-	52.5 ± 0.2
PLA30/PCL70/GNP	156 ± 0.1	63 ± 1.9	124 ± 1.1	1.8 ± 0.1	49.6 ± 0.1
PLA50/PCL50/GNP	153 ± 0.14	61 ± 1.1	124 ± 3.1	1.5 ± 0.05	50.7 ± 0.1
PLA65/PCL35/GNP	148 ± 2.1	58 ± 2.1	115 ± 3.4	7.2 ± 0.2	58.5 ± 0.4
PLA80/PCL20/GNP	155 ± 1.19	63 ± 1.8	132 ± 1.8	1.2 ± 0.04	50.9 ± 0.4
PLA/GNP	155 ± 1.12	-	133 ± 1.7	0.5 ± 0.001	-
PLA	156 ± 2.3	-	-	0.9 ± 0.001	-

**Table 6:** TGA results of PLA, PCL, and their binary and ternary composites.

Sample Name	Maximal Degradation Temperature (T_max_) of Each Polymer		Total Mass Loss (%)	Experimental Percentage of Char Yield (at 600 °C)	Theoretical Percentage of Char Yield (at 600 °C)	Onset Temperature (T_onset_) (°C)
	PLA	PCL				
PCL	-	416 ± 1.6	100 ± 0	0	-	383 ± 2.1
PCL/GNP	-	414 ± 1.7	97 ± 0.01	7.1 ± 0.1	9 ± 0.4	383 ± 1.4
PLA30/PCL70/GNP	361 ± 2	413 ± 3.6	97 ± 0.04	8.1 ± 1.07	9 ± 0.4	351 ± 0.6
PLA50/PCL50/GNP	363 ± 2.1	413 ± 2.2	98 ± 0.02	8.2 ± 0.04	9 ± 0.4	352 ± 0.4
PLA65/PCL35/GNP	364 ± 4.4	412 ± 1.8	95 ± 0.1	14.5 ± 0.9	9 ± 0.4	355 ± 1.1
PLA80/PCL20/GNP	365 ± 1.7	410 ± 4.8	97 ± 0.1	8.1 ± 0.06	9 ± 0.4	351 ± 2
PLA/GNP	368 ± 2.4	-	100 ± 0	4 ± 0.5	9 ± 0.4	351 ± 0.2
PLA	366 ± 4.6	-	100 ± 0	0	-	345 ± 0.6

**Table 7:** Electrical volume resistivity, electric current and LED photos (at 5 v), and PLA continuity percentage of PLA65/PCL35/GNP samples manufactured during different compression moulding times (the PCL continuity was 100% in these samples).

Time (min)	Electrical Volume Resistivity (Ω·cm)	Electric Current (mA) (5 v)	LED (5 v)	Continuous PLA Fraction (%) (Solvent Extraction Method)
10	38.25 ± 8.1	8.56 ± 8 × 10^−2^	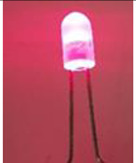	85.247 ± 1.681
20	33.98 ± 9.4	8.89 ± 2 × 10^−2^	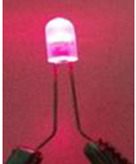	90.527 ± 2.485
35	25.78 ± 5.11	9.24 ± 4 × 10^−2^	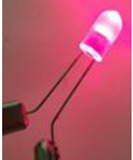	99.481 ± 0.607

**Table 8:** Electrical volume resistivity, electric current and LED photos (at 5 v), and PLA continuity percentage of PLA65/PCL35/GNP samples manufactured using one and two extrusion steps (the PCL continuity was 100% in these samples).

Number of Twin Screw Extrusion Steps	Electrical Volume Resistivity (Ω·cm)	Electric Current (mA) (5 v)	LED (5 v)	Continuous PLA Fraction (%) (Solvent Extraction Method)
One	25.78 ± 5.11	9.24 ± 4 × 10^−2^	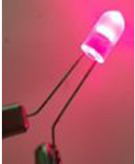	99.481 ± 0.607
Two	30.22 ± 2.81	9.05 ± 6 × 10^−2^	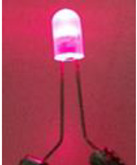	95.843 ± 0.759

**Table 9:** Electrical volume resistivity and electric current results with the accompanied LED photos at 5 v of PLA65/PCL35/GNP composites (GNP content varies between 5 wt.% and 20 wt.%).

Weight Percentage of Graphene (%)	Electrical Volume Resistivity (Ω·cm)	Electric Current (mA) (5 v)	LED (5 v)
5	486.5 ± 10	18 × 10^−3^ ± 5 × 10^−3^	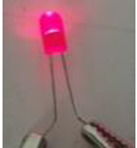
10	25.78 ± 5.11	9.24 ± 4 × 10^−2^	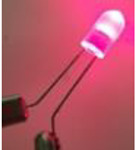
15	12 × 10^−2^ ± 3 × 10^−3^	15.65 ± 1.94	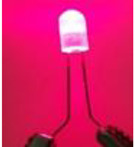
20	2 × 10^−3^ ± 6 × 10^−4^	25.55 ± 6.88	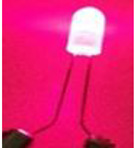

**Table 10:** Electrical volume resistivity and electric current results with the accompanied LED photos at 5 v of the 3D-printed and compression-moulded samples containing 10 wt.% GNP and PLA content varying between 80 wt.% and 30 wt.%.

Sample	Electrical Volume Resistivity (3D Printed) (Ω·cm)	Electric Current (3D Printed) (mA) (5 v)	LED (3D Printed) (5 v)	Electrical Volume Resistivity (Compression Moulded) (Ω·cm)	Electric Current (Compression Moulded) (mA) (5 v)	LED (Compression Moulded) (5 v)
PLA80/PCL20/GNP	>10^7^	-	No light	4900.43 ± 90.19	10^−6^ ± 4 × 10^−3^	No light
PLA65/PCL35/GNP	1000.53 ± 30.22	10^−3^ ± 10^−2^	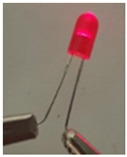	25.78 ± 5.11	9.24 ± 4 × 10^−2^	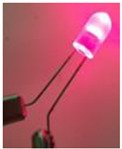
PLA50/PCL50/GNP	3277.83 ± 89.32	9 × 10^−6^ ± 5 × 10^−5^	No light	75.67 ± 15.19	3.1 ± 5 × 10^−2^	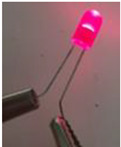
PLA40/PCL60/GNP	5371.62 ± 90.44	6 × 10^−6^ ± 7 × 10^−5^	No light	125.61 ± 20.81	1.4 ± 2 × 10^−2^	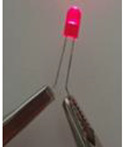
PLA30/PCL70/GNP	7000 ± 101.34	10^−7^ ± 9 × 10^−5^	No light	237.34 ± 25.33	0.9 ± 10^−2^	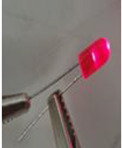

## Data Availability

The data presented in this study are available on request from the corresponding author.
